# Metabolome-Based Discrimination Analysis of Five *Lilium* Bulbs Associated with Differences in Secondary Metabolites

**DOI:** 10.3390/molecules26051340

**Published:** 2021-03-02

**Authors:** Ying Kong, Huan Wang, Lixin Lang, Xiaoying Dou, Jinrong Bai

**Affiliations:** 1 Beijing Radiation Center, Beijing 100875, China; kongying@brc.ac.cn (Y.K.); wanghuan@brc.ac.cn (H.W.); langlixin@brc.ac.cn (L.L.); douxiaoying@brc.ac.cn (X.D.); 2 Key Lab of Beam Technology and Material Modification of Ministry of Education, College of Nuclear Science and Technology, Beijing Normal University, Beijing 100875, China

**Keywords:** lily, metabolomics analysis, saponin, flavonoid, phenolic acid

## Abstract

The bulbs of several *Lilium* species are considered to be both functional foods and traditional medicine in northern and eastern Asia. Considering the limited information regarding the specific bioactive compounds contributing to the functional properties of these bulbs, we compared the secondary metabolites of ten *Lilium* bulb samples belonging to five different species, using an ultrahigh-performance liquid chromatography-electrospray ionization-tandem mass spectrometry (UPLC-ESI-MS/MS)-based secondary metabolomics approach. In total, 245 secondary metabolites were detected; further, more metabolites were detected from purple *Lilium* bulbs (217 compounds) than from white bulbs (123–171 compounds). Similar metabolite profiles were detected in samples within the same species irrespective of where they were collected. By combining herbal analysis and screening differential metabolites, steroid saponins were considered the key bioactive compounds in medicinal lilies. Of the 14 saponins detected, none were accumulated in the bulbs of *L*. *davidii* var. *willmottiae,* also called sweet lily. The purple bulbs of *L*. *regale* accumulated more secondary metabolites, and, notably, more phenolic acid compounds and flavonoids. Overall, this study elucidates the differential metabolites in lily bulbs with varying functions and colors and provides a reference for further research on functional foods and the medicinal efficacy of *Lilium* species.

## 1. Introduction

The genus *Lilium* comprises more than 100 species worldwide, and China is the main distribution center of *Lilium,* with 55 species [1,2]. The lily bulb, without its enveloping protective sheath, is composed of firm, fleshy scales that provide nourishment for the developing plant. The scales are modified leaves that are thickened and shortened to act as a store of food reserves [3]. The carbohydrates stored by lily plants include starch, sugar, and so on. The starch content accounts for 11–22% of the bulb’s fresh weight (Table 1), and intrinsic free sugar content, mainly including sucrose, glucose, and fructose, accounts for 2–14% of the fresh weight [4]. 

In addition to providing nutrients to the plant itself, the lily bulb has accumulated a variety of secondary metabolites serving as defenses for plants, including major groups such as phenolics, terpenoids, and nitrogen-containing compounds [5]. The ancient Chinese people discovered the medicinal properties of lily bulbs and recorded them in ancient medical books. The earliest known record of lily bulb efficacy is found in the book Shen Nong Ben Cao Jing, which was compiled before the second century AD [6]. Nowadays, the dry scales of three *Lilium* bulbs, including *L*. *pumilum* (*Shan Dan*, SD), *L*. *lancifolium* (*Juan Dan*, JD), and *L*. *brownii* var. *viridulum* (*Bai He*, and *Longya Baihe* for trade name, LY), have been officially listed in the Chinese Pharmacopeia (2020 edition) and are considered medicative for bronchitis, pneumonia, and cough [7]. Many natural products have been separated and identified from lily bulbs, including phenylpropenoid glycerides [8,9], phenolic compounds [10], and steroidal saponins [11], which exhibit excellent antioxidant, antibacterial, and anti-inflammatory effects [12]. However, the specific bioactive compounds responsible for the efficacy of lily bulbs remain unclear.

In addition to its medicinal uses, the lily bulb is also considered a good root vegetable that provides carbohydrates, proteins, and a low fat content [13]. Lily bulbs can be used for daily consumption and are thus known as medicinal and edible (M&E) plants. According to the traditional theories of Chinese medicine, eating lily bulbs is beneficial as they include nourishing yin, moisten the lungs, and clear heartburn [9]. Aside from the three abovementioned *Lilium* species, the bulbs of *L*. *davidii* var. *willmottiae* (*Lanzhou Baihe*, LZ) are also popular products in Chinese vegetable markets, and are called ”sweet lily bulbs” [14]. In contrast, JD is called a “bitter lily bulb.” Their different tastes may result from the differential accumulation of secondary metabolites because many plant-based phenols and flavonoids are bitter or astringent [15]. Some bitter substances have been isolated from other *Lilium* bulbs, mainly phenylpropanoid glycosides and derivatives, including regaloside A and B, and some ferulic acid esters of sucrose [16,17,18,19]. Whether there are other substances that contribute to the bitterness of lily is unclear.

These four lily bulbs that are commonly used as edible and as medicine are mostly light-colored. In nature, *Lilium* bulbs exist in a wide range of color traits from white to yellow and deep purple. When exposed to light, many light-colored lily bulbs take on a rosy-purple coloration [20]. Even closely related *Lilium* species may have different bulb colors. For example, LY and *L*. *regale* (*Minjiang Baihe*, MJ), both of which possess trumpet white flowers, have mature bulbs that are white and purple, respectively. Nowadays, it is generally believed that dark-colored vegetables contain more bioactive phytochemicals, such as flavonoids and anthocyanins. Consequently, pigmented root vegetables such as sweet potatoes have attracted increased attention from scientists and consumers [21]. However, the purple MJ bulbs are not considered edible because of their highly bitter taste [13]. Overall, purple lilies are speculated to accumulate more anthocyanins. However, whether they contain other differential metabolites remains unclear. 

Therefore, the objectives of this study were (1) to screen out the key active metabolites in medicinal lily bulbs, (2) to discuss the reasons for the difference in taste between sweet and bitter lily bulbs, and (3) to explore the differential secondary metabolites between deep purple lily bulbs and white lily bulbs. This study provides a comprehensive understanding of the bioactive natural compounds in lily bulbs, and it is beneficial to the further development and utilization of secondary metabolites in *Lilium* species.

## 2. Results and Discussion

### 2.1. Metabolite Profiling Analysis of Five Lilium Bulbs

**Figure 1:**
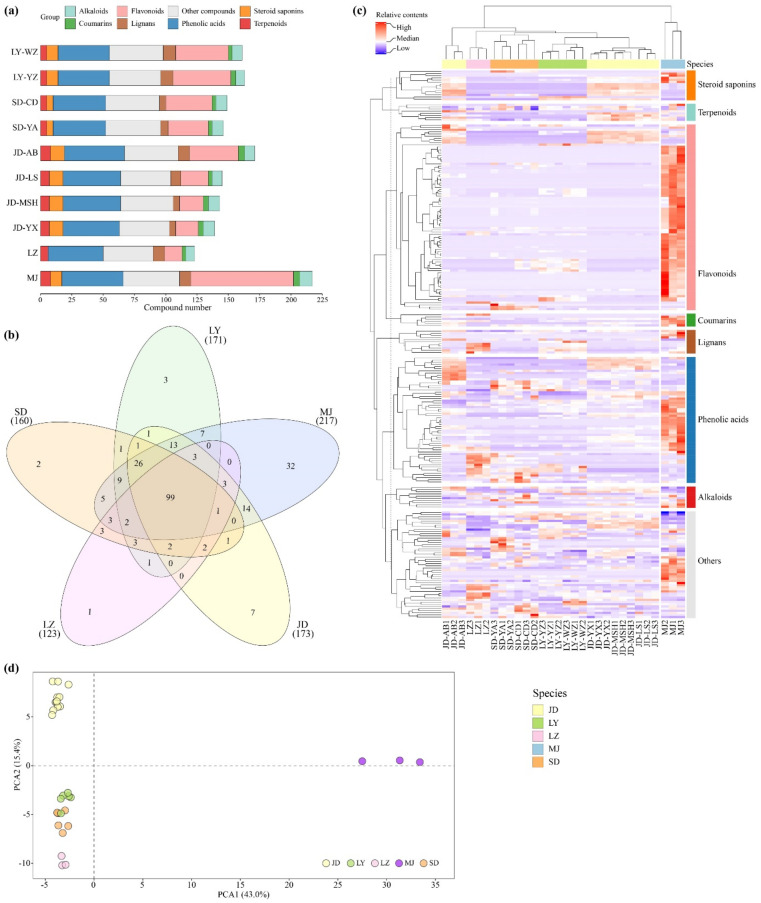
Secondary metabolite profiles of ten lily samples belonging to five different species. (**a**) The number of secondary metabolites detected in ten lily samples; (**b**) Venn diagrams for displaying the number of commonly and specific metabolites among five *Lilium* species; (**c**) Hierarchical cluster analysis and heatmap of metabolite profiles of ten lily samples. Clustering distance method ”Pearson”, hierarchical clustering method “complete”; (**d**) Principal component analysis and scores plot for ten lily samples.

In total, 245 metabolites (87 flavonoids, 59 phenolic acids, 14 steroids, 10 alkaloids, 6 coumarins, 11 lignans, 8 terpenoids, and 50 other compounds) were identified or annotated in all ten lily samples. Most of the metabolites were detected in the purple bulbs of MJ (217 compounds), whereas eight lily samples belonging to three M&E *Lilium* species accumulated fewer secondary metabolites (139–171 compounds). The number of secondary metabolites in the edible LZ samples was the least (123 compounds) (Figure 1a). Compared with the three cultivated JD samples (JD-LS, JD-MSH, and JD-YX), more secondary metabolites were accumulated in samples of JD-AB (Figure 1a), which grew at high altitudes (about 2000 m) in the wild environment. Ninety-nine common metabolites were detected in all lily bulbs, and the correlation coefficients of white lily bulbs were high (Pearson’s *r* > 0.7) (Figure S1, Supplementary Materials), possibly because light-colored lily samples had fewer metabolites, whereas purple MJ bulbs accumulated more specific metabolites (Figure 1b).

Both hierarchical cluster analysis (HCA) and principal component analysis (PCA) were performed, and the edible lilies with white bulbs were separated from MJ with purple bulbs. The relative contents of many metabolites were higher in MJ samples (Figure 1c,d). The results showed that samples within the same species clustered together except for JD-AB (Figure 1c). However, the results of PCA plots indicated that except for a sample of LY bulbs which was closer to the SD group, other samples were distributed together by species (Figure 1d). This suggested that samples within the same species demonstrated similar metabolite profiles regardless of where they were planted. Overall, each *Lilium* species was found to possess its own unique and distinguishable metabolite profile.

### 2.2. Differential Metabolite Analysis in Medical Lily Bulbs

**Figure 2:**
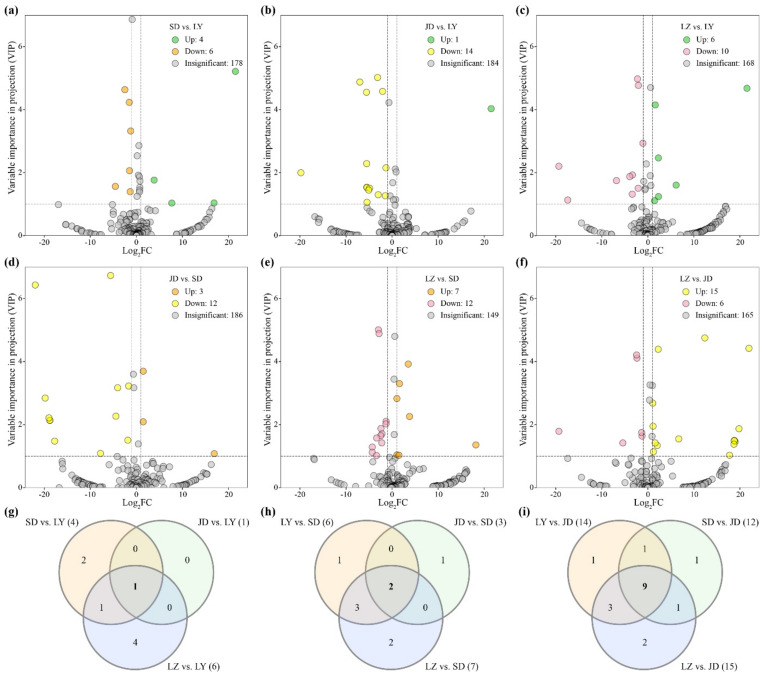
Differential metabolites in four edible *Lilium* bulbs. Upregulated metabolites are indicated in green (*L. brownii* var. *viridulum*, LY), orange (*L. pumilum,* SD), yellow (*L. lancifolium*, JD), and pink (*L. davidii* var. *willmottiae*, LZ). (**a**) Volcano plot of the differential metabolites between SD and LY. (**b**) Volcano plot of the differential metabolites between JD and LY. (**c**) Volcano plot of the differential metabolites between LZ and LY. (**d**) Volcano plot of the differential metabolites between JD and SD. (**e**) Volcano plot of the differential metabolites between LZ and SD. (**f**) Volcano plot of the differential metabolites between LZ and JD. (**g**) Venn plot indicating the number (in bold) of commonly upregulated metabolites among the comparison groups of SD vs. LY, JD vs. LY, and LZ vs. LY. (**h**) Venn plot indicating the number (in bold) of commonly upregulated metabolites among the comparison groups of LY vs. SD, JD vs. SD, and LZ vs. SD. (**i**) Venn plot indicating the number (in bold) of commonly upregulated metabolites among the comparison groups of SD vs. LY, JD vs. LY, and LZ vs. LY.

By comparing the relative metabolite contents between four edible *Lilium* species, the upregulated (log_2_FC (fold change) ≥1, VIP ≥ 1) and downregulated (log_2_FC ≤ −1, VIP ≥ 1) metabolites were screened (Figure 2a–f). Based on the speculation that higher bioactive ingredients were accumulated in medicinal lilies, the commonly upregulated metabolites in each medicinal *Lilium* species were chosen and analyzed (Figure 2g–i).

#### 2.2.1. Differential Metabolites in LY Bulbs

Although the bulbs of three *Lilium* species were considered medicative, recent systematic studies on their origin and quality based on textual research and investigation indicated that *Lilium* plants with white trumpet flowers and white bulbs are an authentic medicinal material [6,22,23]. Therefore, the bulbs of *L*. *brownii* and *L*. *brownii* var. *viridulum*, which were the most common and widespread species at that time, were considered to be of good quality in the ancient herbal literature (Figure S2). 

**Figure 3:**
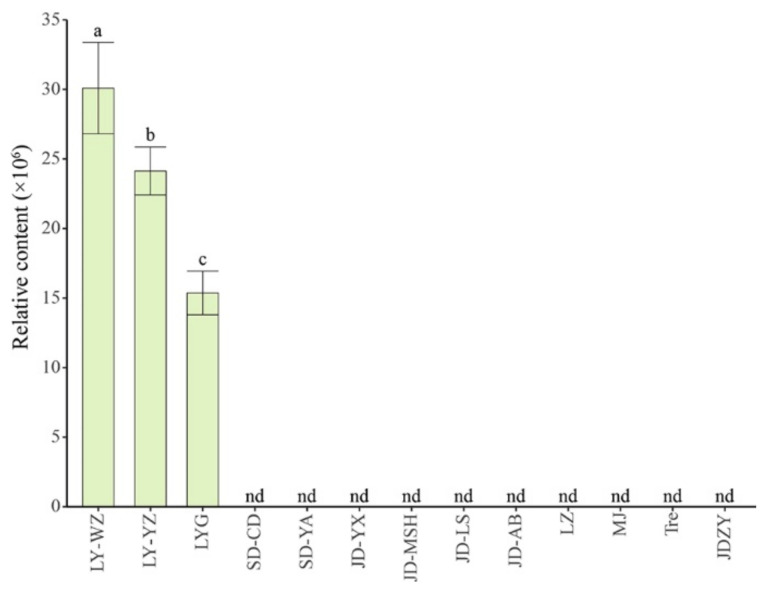
The relative contents of a steroidal saponin that was temporarily identified as 26-*O*-glucopyranosyl-furost-5-3,26-diol 3-*O*-[rhamnopyranosyl-(1→2)]-glucopyranoside in different lily samples. Data are presented as the mean ± standard error (SE, *n* = 3). Different lowercase letters indicate statistically significant differences (*p* < 0.05). LYG: dry scales of LY bulbs, Tre: bulbs of *Lilium* “Tresor”, JDZY: bulbils of *L*. *lancifolium*. nd: not detected.

There were four upregulated metabolites between SD and LY (Figure 2a), only one upregulated metabolite between JD and LY (Figure 2b), and six upregulated metabolites between LZ and LY (Figure 2c). In total, there was only one commonly differential metabolite in LY (Figure 2g), which was not detected in other lily samples; this was a steroidal saponin with the molecular formula C_45_H_74_O_18_ (MW 902.43), which was temporarily identified as 26-*O*-glucopyranosyl-furost-5-3,26-diol 3-*O*-[rhamnopyranosyl-(1→2)]-glucopyranoside [11]. Combined with the relative amounts of this compound in three other lily samples in our pre-experiment, including the dry scales of LY, the bulbils of JD-AB, and the bulbs of *Lilium* ‘Tresor’, we found that this steroidal saponin was exclusively detected in LY scales regardless of whether they were fresh or dried (Figure 3). As steroidal saponins have multiple spatial configurations and isomers in *L*. *brownii* [24,25], it is difficult to determine their specific structure based only on the MS spectrum (Figure S3). Because some saponins were not detected [26], we speculated that steroidal saponins may be the key bioactive metabolites in medicinal lily bulbs.

#### 2.2.2. Differential Metabolites in SD Bulbs

Although SD is also used as a medicinal plant, its efficacy differs from that of LY. The traditional pharmacopoeia records indicate that SD can be used for treating various skin conditions including bruises, swelling, and wounds. There were two commonly upregulated differential metabolites among the comparison groups LY vs. SD, JD vs. SD, and LZ vs. SD (Figure 2a,d,e,h); 1,2-*O*-diferuloylglycerol and 1,3-*O*-diferuloyl*-*glycerol were both detected in all lily samples in this study. However, after one-way analysis of variance (ANOVA) with other samples, their content difference between SD and some JD samples was not significant (Figure S4). These two ferulic acid derivatives with antioxidant and ultraviolet light-absorbing properties accumulated slightly higher only in the SD samples [8,9,27]; thus, further studies are needed to determine whether they are the key active components in SD.

#### 2.2.3. Differential Metabolites in JD Bulbs

Similarly, there were nine commonly upregulated differential metabolites found in the JD samples (Figure 2b,d,f,i). After excluding compounds that were not significantly different from other lily samples, eight metabolites were screened out, including one flavonoid, three phenolic compounds, three steroids, and one coumarin (Figure S5). The easily identified JD has orange flowers with black spots and bulbils, and the Ben Cao Tu Jing (Illustrated Pharmacopoeia), which dates from about 1080, clearly mentions that “it is not suitable for medicinal use” [6]. Subsequent pharmacy books have also recorded that JD does not have the same medicinal effects as LY. However, in Korea, the bulbs of JD are used to treat bronchitis, pneumonia, and so on [28]. JD bulbs are also reported to possess potent antioxidant and anticancer characteristics [8,29]. Other medicinal effects of JD remain to be studied.

### 2.3. Differential Metabolites Analysis Sweet and Bitter Lily Bulbs

The taste description of LY and SD bulbs is controversial; therefore, we chose JD and LZ samples to represent bitter and sweet lily bulbs, respectively. Notably, no steroidal saponins were detected in LZ samples in this study (Figure 1a). After screening the differential metabolites (Figure 2f) and excluding the metabolites with insignificant differences between LZ and JD, there were 12 upregulated differential metabolites including 5 phenolic acids, 4 steroids, 1 flavonoid, 1 coumarin, and 1 other compound, and 6 downregulated differential metabolites, including 3 phenolic acids, 1 flavonoid, and 2 other compounds (Figure S6). 

Although many phenolic compounds are bitter substances, both JD and LZ accumulated differential phenolic acids, and it was difficult to distinguish their contribution to the bitter taste of lily bulbs. Moreover, some saponins also have a bitter taste [30]. A previous study found that the taste of quinoa varieties (*Chenopodium quinoa*), bitter or sweet, was related to their saponin contents [31], and that sweeteners such as sugars were commonly used agents to reduce perceived bitterness [32]. Our previous research found that the intrinsic free sugar content in LZ bulbs was higher (11.2–11.7%) than that in JD bulbs (5.1–7.6%) [4]. Therefore, we speculated that the bitter taste of JD bulbs might relate to their low content of free sugars and high accumulation of saponins. 

### 2.4. Differential Metabolite Analysis between Purple and White Lily Bulbs

**Figure 4:**
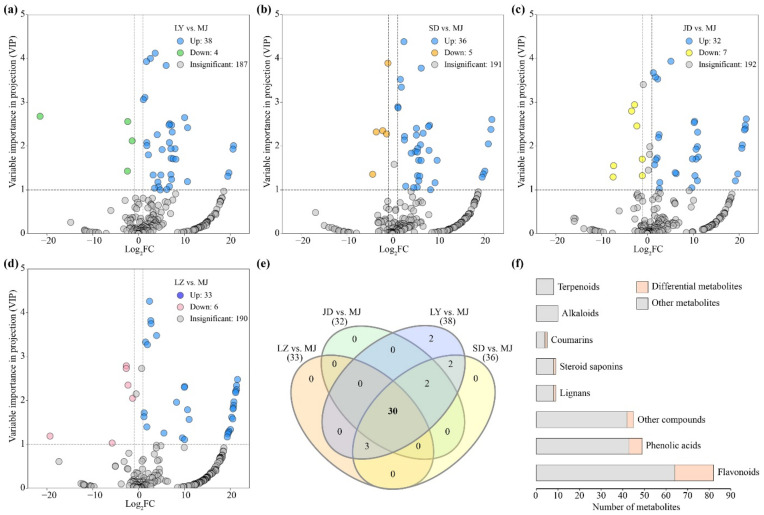
Differential metabolites between purple *L*. *regale* (MJ) bulbs and edible white bulbs. Upregulated metabolites are indicated in blue (MJ). (**a**) Volcano plot of the differential metabolites between *L*. *brownii* var. *viridulum* (LY) and MJ. (**b**) Volcano plot of the differential metabolites between *L*. *pumilum* (SD) and MJ. (**c**) Volcano plot of the differential metabolites between *L*. *lancifolium* (JD) and MJ. (**d**) Volcano plot of the differential metabolites between *L*. *davidii* var. *willmottiae* (LZ) and MJ. (**e**) Venn plot indicating the number (in bold) of commonly upregulated metabolites in MJ samples. (**f**) The number of differential metabolites in different compound groups.

**Figure 5:**
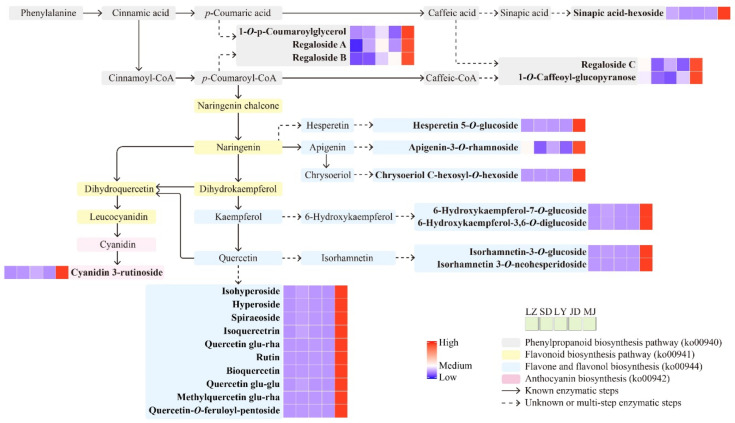
Upregulated differential phenolic acids and flavonoids (in bold) between purple *L*. *regale* (MJ) bulbs and edible white bulbs were mapped to the metabolic pathway networks. LZ: *L*. *davidii* var. *willmottiae*, SD: *L*. *pumilum*, LY: *L*. *brownii* var. *viridulum*, JD: *L*. *lancifolium*.

In addition to more metabolites being detected in the purple bulbs of MJ, 32 metabolites were exclusively detected in these bulbs (Figure 2b). Combining the relative contents and orthogonal partial least square discriminant analysis (OPLS-DA) results with four edible lily samples (log_2_FC ≥ 1, VIP ≥ 1), we found 38 upregulated metabolites between LY and MJ (Figure 4a), 36 upregulated metabolites between SD and MJ (Figure 4b), 32 upregulated metabolites between JD and MJ (Figure 4c), and 33 upregulated metabolites between LZ and MJ (Figure 4d). In total, there were 30 commonly upregulated differential metabolites between purple MJ bulbs and edible white bulbs (Figure 4e, Table S1), including 18 flavonoids, 6 phenolic acids, 1 coumarin, 1 lignan, 1 steroid, and 3 other compounds (Figure 4f). These upregulated differential metabolites in MJ bulbs were mapped to the KEGG database to analyze their biochemical synthesis pathways. The metabolic pathways found to be differentially altered between the purple and white lily bulbs mainly included phenylpropanoid biosynthesis (ko00940), flavonoid biosynthesis (ko00941), flavone and flavonol biosynthesis (ko00944), and anthocyanin biosynthesis (ko00942) (Figure 5).

Five of the six phenolic acid compounds were phenolic glycerides and their derivatives. The synthesis pathway of these phenolic glycerides (glycosides) is still unclear. However, it is speculated that they might be catalyzed by glycerol *O*-hydroxycinnamoyltransferase or glycerol transferase and then by glycosyltransferase, of which the former can employ *p*-coumaroyl-CoA or caffeoyl-CoA as an acyl donor and use glycerol as an acceptor [33].

Among these differential flavonoid metabolites, the relative contents of cyanidin 3-rutinoside, the main anthocyanin compound, were higher in the MJ samples as expected (Figure 5), which is also a major component of red and pink lily flowers [34]. In addition, 14 of 18 differential flavonoids are flavonol glycosides, including 2 kaempferol derivates and 12 quercetin derivates. This is consistent with previous research showing that the quercetin and rutin contents in MJ bulbs were significantly higher than those in SD, JD, and LZ bulbs [10]. Although quercetin and its derivatives are pharmacologically active and have various biological activities, their bitter flavors, which contribute to the bitter taste of MJ bulbs, make them unpopular [35]. Although studies are underway to obtain functional foods with more biological activities and improved taste, introducing these bioactive substances from MJ bulbs into edible lilies is a topic worthy of further in-depth study.

## 3. Materials and Methods

### 3.1. Plant Material

**Figure 6:**
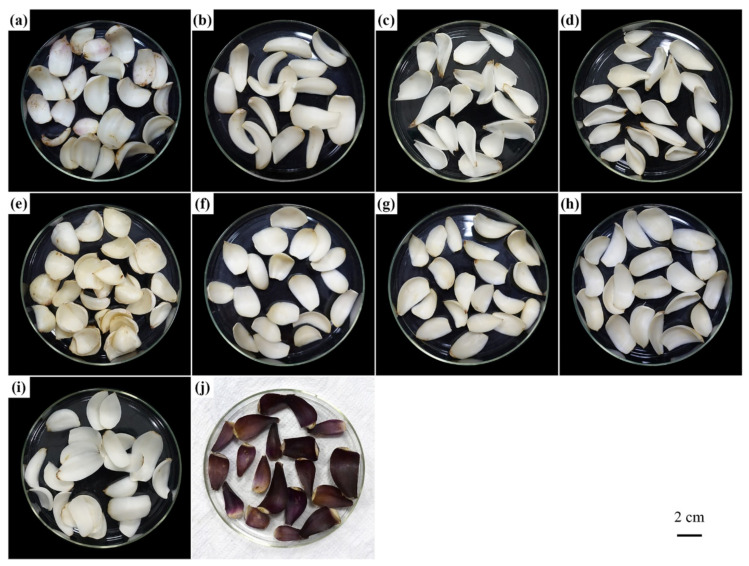
Photos of ten *Lilium* bulb samples belonging to five different species. (**a**) LY-WZ; (**b**) LY-YZ; (**c**) SD-CD; (**d**) SD-YA; (**e**) JD-AB; (**f**) JD-LS; (**g**) JD-MSH; (**h**) JD-YX; (**i**) LZ; (**j**) MJ.

Light-colored bulbs of four edible *Lilium* species were collected, mainly from their natural habitats. Bulbs of *L*. *regale*, which were propagated from seeds, collected from Sichuan Province and planted in the Lily Germplasm Resource Preservation Centre (116^°^43′ N, 40^°^16′ E) for three to four years, were also used. Samples were collected from September to October 2019, and their information is listed in Table 1, Figure 6, and Figure S2.

Fresh bulbs of *Lilium* species were first stored at −4°C for one week and then washed and dried at room temperature for 15 min. Three bulbs were used for each sample. The scales were cut into 1 × 1 cm^2^ pieces and frozen in liquid nitrogen. After freeze-drying (SCIENTZ-50ND, SCIENTZ), the samples were ground into a powder using a mixer mill (MM 400, Retsch) with zirconia beads for 1.5 min at 30 Hz, and then stored at −80°C until extraction.

### 3.2. Reagents

Methanol and acetonitrile (HPLC grade) were purchased from Merck (Darmstadt, Germany). Acetic acid was obtained from Sigma (St. Louis, MO, USA). MilliQ water (Millipore, Bradford, PA, USA) was used in all experiments. 

### 3.3. Sample Extraction

A fine powder sample of a dried lily bulb (100 mg) was extracted overnight at 4 ℃ using 1.0 mL of 70% aqueous methanol. The homogenate was centrifuged at 10,000× *g* for 10 min, and the extracts were cleaned up by solid-phase extraction (SPE, CNWBOND Carbon-GCB cartridge, 250 mg, 3 mL; ANPEL, Shanghai, China) and were filtrated (SCAA-104, 0.22 μm pore size; ANPEL, Shanghai, China) before LC–MS analysis. 

### 3.4. HPLC–MS/MS Analysis

Samples were injected into an LC–ESI–MS/MS system (HPLC, Shim-pack UFLC SHIMADZU CBM30A system; MS, Applied Biosystems 4500 Q TRAP). The analytical conditions were as follows, HPLC: column, Waters ACQUITY UPLC HSS T3 C18 column (1.8 µm, 2.1 mm × 100 mm); solvent system, ultrapure water (Solvent A, 0.04% acetic acid): acetonitrile (Solvent B, 0.04% acetic acid); gradient program, 100/0, 5/11.0, 5/12.0, 95/12.1, 95/15.0 (min/% A, *v*/*v*), flow rate, 0.40 mL/min; temperature, 40 °C; injection volume: 5 μL. The effluent was alternatively connected to an ESI–triple quadrupole–linear ion trap (Q TRAP)–MS system. The ESI–Q TRAP–MS/MS analytical conditions used were as reported by Chu et al. [36]. 

### 3.5. Metabolite Annotation and Quantification

Metabolite identification was based on the primary and secondary spectrometry data, annotated and compared against public databases, including MassBank, KNApSAcK, HMDB, METLIN, and the MWDB Database (MetWare Biological Science and Technology Co. Ltd., Wuhan, China), following the standard metabolic operating procedures. The phenolic compounds were divided into different subgroups based on their chemical structures, such as phenolic acids, flavonoids, coumarins, and lignans, as reported by Gan et al. [37]. 

The metabolites were quantified via the multiple reaction monitoring mode (MRM) using triple quadrupole mass spectrometry as reported by Wang et al. [38]. After obtaining metabolite data from different samples, the peak area of the mass spectra of all metabolites was integrated, and the mass spectra of the same metabolites in different samples were integrated and corrected.

### 3.6. Screening of Differential Metabolites

Hierarchical cluster analysis (HCA), principal component analysis (PCA), and correlation analysis were performed using the ComplexHeatmap, factoextra, and corrplot packages in RStudio, respectively [39]. For HCA, after normalizing the signal intensities of each metabolite (unit variance scaling), the results of the samples and metabolites were presented as heatmaps with dendrograms. After data were subjected to Pareto scaling by SIMCA software (V14.1, MKS Data Analytics Solutions, Sweden), orthogonal partial least square discriminant analysis (OPLS-DA) was performed, and the validity of the OPLS-DA model was evaluated [40]. The variable importance in projection (VIP) of metabolites was calculated for the subsequent screening of differential metabolites. To compare the differential metabolite variation, Venn diagrams were plotted using the InteractiVenn tool [41]. Significant differences (*p* < 0.05) were determined by one-way analysis of variance (ANOVA) using SPSS 23 (SPSS Inc., Chicago, IL, USA). Differential metabolites were mapped to the Kyoto Encyclopaedia of Genes and Genomes (KEGG) database (www.kegg.jp/kegg/pathway.html, accessed on 15 January 2021) for metabolic network analysis.

## 4. Conclusions

The bulbs of several *Lilium* species are esteemed as culinary delicacies in northern and eastern Asia and are also considered to have medicinal properties. In this study, we analyzed the secondary metabolites of 10 lily bulb samples from different habitats, belonging to five species. In total, 245 metabolites (including 87 flavonoids, 59 phenolic acids, 14 steroids, 10 alkaloids, 6 coumarins, 11 lignans, 8 terpenoids, and 50 other compounds) were identified. Combined literature analysis and metabolomic analysis suggested that *L*. *brownii* var. *viridulum* was considered the authentic lily for traditional medical use and that steroid saponins may be the key active metabolites in medicinal lily bulbs. The bitter taste of *L*. *lancifolium* might be related to its low content of free sugars and high accumulation of saponins. The purple bulbs of *L*. *regale* showed a differential accumulation of 30 metabolites, including 18 flavonoids and 6 phenolic acids. Overall, this study elucidates the differences in secondary metabolites among different *Lilium* bulbs, provides a reference for the utilization of and research on lilies as plant medicine and functional food, and may guide the development and genetic enhancement of functional lily bulb varieties.

## Figures and Tables

**Table 1:** Information of ten *Lilium* samples.

Name	Application ^a^	Section ^b^	Bulb Color	Province	Location	Code	Circumference (cm) ^c^	Starch Content (%)
*L*. *brownii* var. *viridulum*	M&E	L	White	Jiangxi	Wanzai	LY-WZ	19.80 ± 1.17	22.80
				Hunan	Yongzhou	LY-YZ	23.13 ± 0.38	/
*L. pumilum*	M&E	S	White	Hebei	Chengde	SD-CD	7.27 ± 0.13	14.55
				Shaanxi	Yan’an	SD-YA	6.73 ± 0.33	12.06
*L*. *lancifolium*	M&E	S	White	Sichuan	A’ba	JD-AB	13.80 ± 0.31	19.16
				Hunan	Longshan	JD-LS	25.97 ± 0.15 ^d^	15.82
				Anhui	Manshuihe	JD-MSH	22.50 ± 0.26 ^d^	11.86
				Jiangsu	Yixing	JD-YX	27.03 ± 0.69 ^d^	17.99
*L*. *davidii* var. *willmottiae*	E	S	White	Gansu	Lanzhou	LZ	15.97 ± 0.26	11.64
*L*. *regale*	/	L	Purple	Beijing	Huairou	MJ	14.40 ± 0.87	13.33

^a^ M, medicinal; E, edible. ^b^ L, Section *Leucolirion*; S, Section *Sinomartagon*. ^c^ Values present mean ± standard error (SE) of triplicate tests. ^d^ The bulbs of cultivated *L*. *lancifolium* are connate with 4–5 smaller bulbs.

## Data Availability

Data presented are available in the manuscript and Supplementary Materials.

## Supplementary Materials

The following are available online. Figure S1: Correlation analysis on the metabolite profiles of ten lily samples, Figure S2: The distribution area and collection place of ten lily samples belonging to five different species, Figure S3: The MS/MS spectrum of the [M-H]^+^ ion of 26-*O*-glucopyranosyl-furost-5-3,26-diol 3-*O*-[rhamnopyranosyl-(1→2)]-glucopyranoside, Figure S4: The relative contents of upregulated metabolites in SD samples compared with other edible lily samples, Figure S5: The relative contents of upregulated metabolites in JD samples compared with other edible lily samples, Figure S6. The relative contents of differential metabolites in JD and LZ samples. Table S1: A list of upregulated metabolites identified between the purple and white lily bulbs.

## Sample Availability

Samples of the compounds are not available from the authors.
