# Supplementary material for: Metabolome-Based Discrimination Analysis of Five Lilium Bulbs Associated with Differences in Secondary Metabolites
Source: Molecules. 2021 Mar 2;26(5):1340. doi: 10.3390/molecules26051340 (PMC7958954; doi:10.3390/molecules26051340)
Supplement: Supplementary file 1 [file molecules-26-01340-s001.pdf]

Supplementary Materials

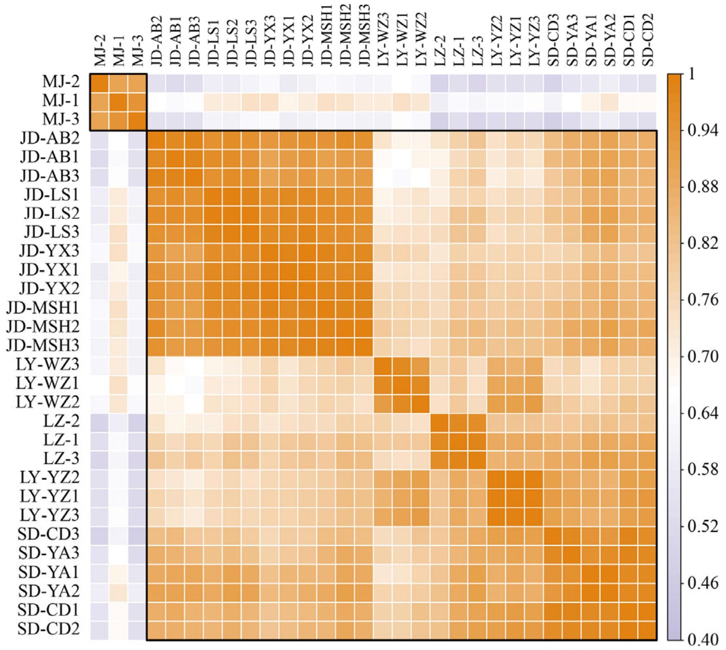

Figure S1. Correlation analysis on the metabolite profiles of ten lily samples.

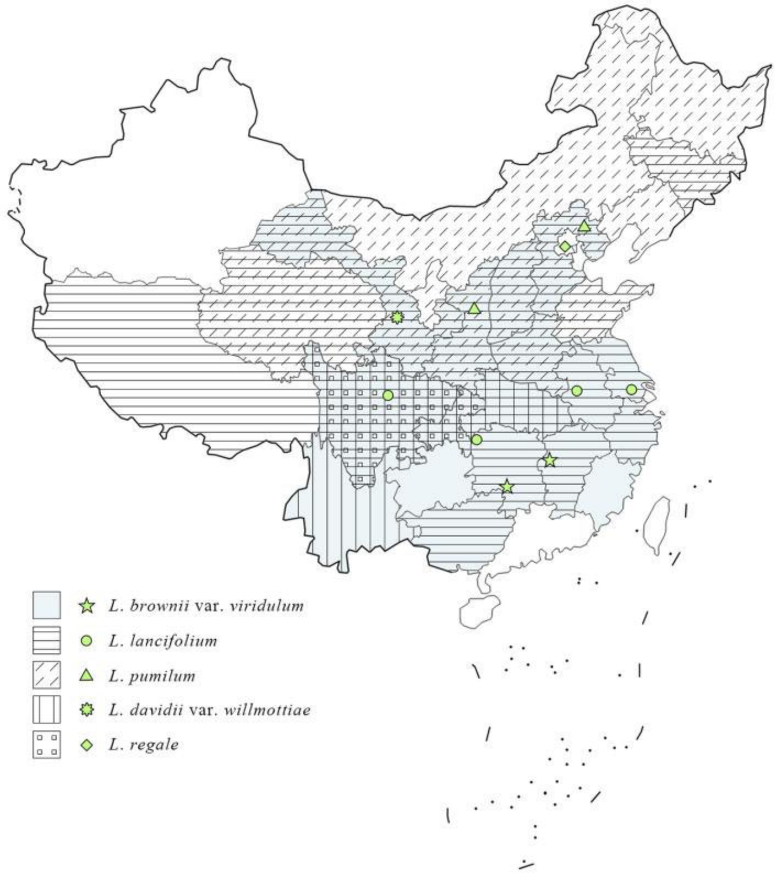

Figure S2. The distribution area and collection place of ten lily samples belonging to five different species.

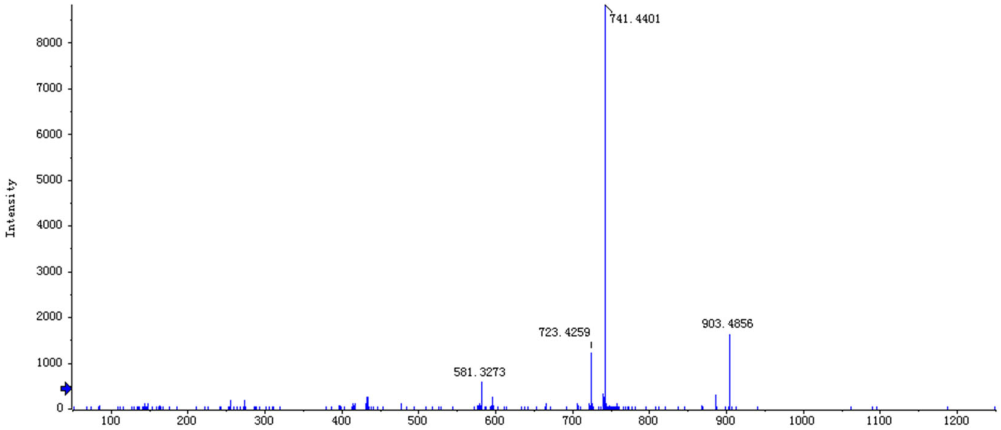

Figure S3. The MS/MS spectrum of the [M-H]⁺ ion of 26-*O*-glucopyranosyl-furost-5-3,26-diol 3-*O*-[rhamnopyranosyl-(1→2)]-glucopyranoside.

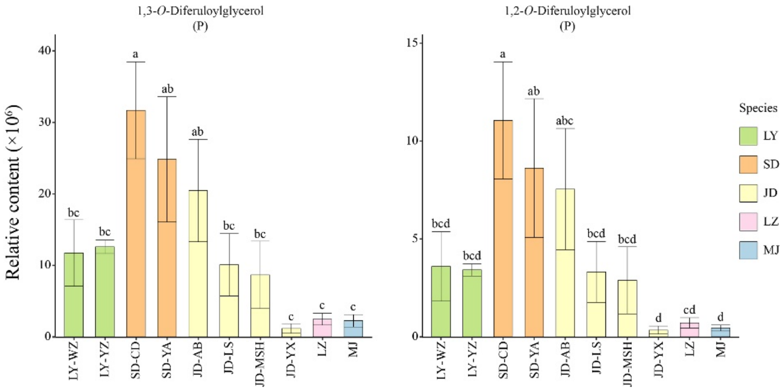

Figure S4. The relative contents of upregulated metabolites in *Lilium pumilum* (SD) samples compared with other edible lily samples. The relative contents of *L. regale* (MJ) samples were also listed. Data are presented as the mean ± standard error (SE, $n = 3$). Different lowercase letters indicate statistically significant differences ($P < 0.05$). LY: *L. brownii* var. *viridulum*, JD: *L. lancifolium*, LZ: *L. davidii* var. *willmottiae*. P: phenolic acids.

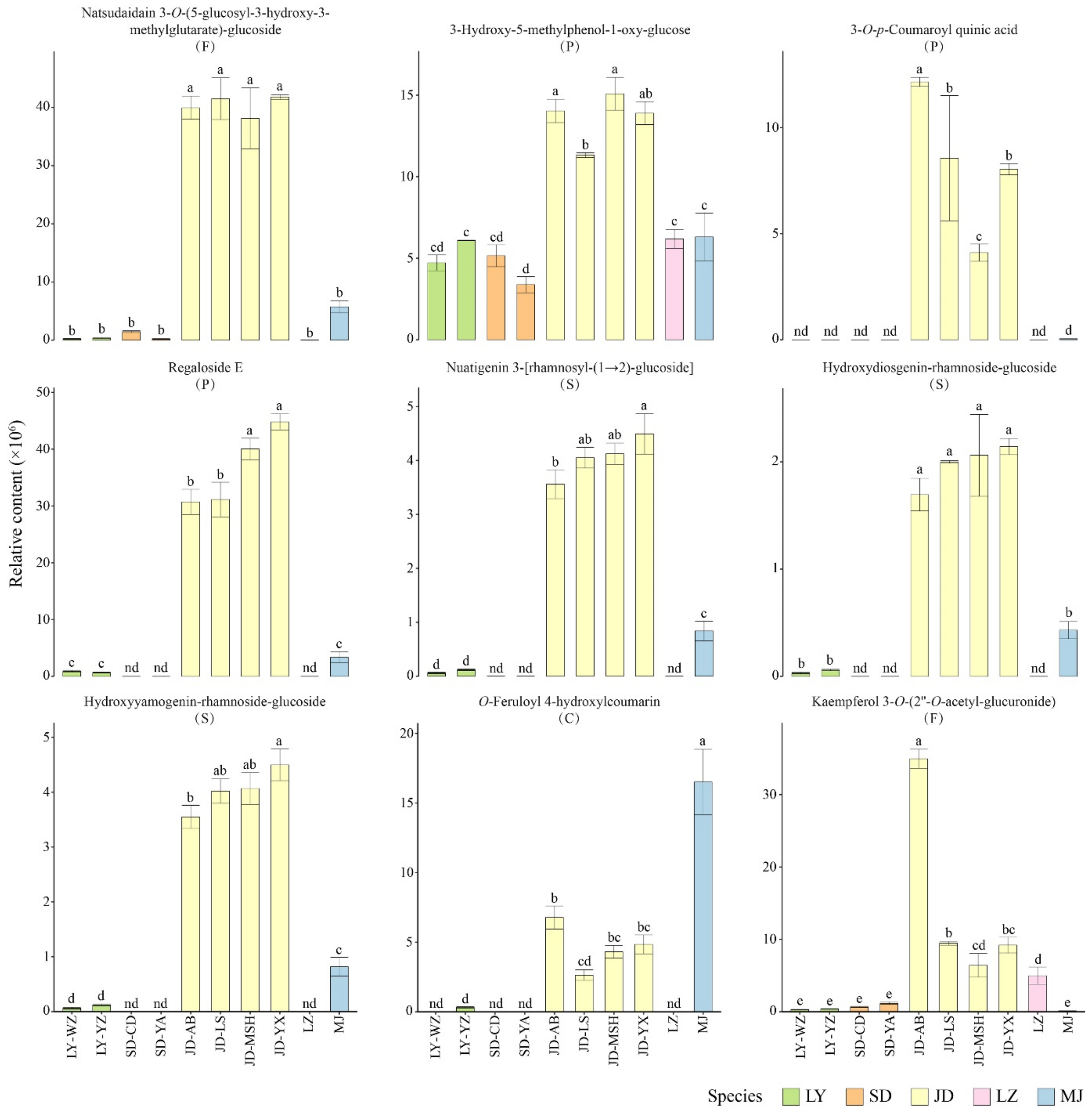

Figure S5. The relative contents of upregulated metabolites in *L. lancifolium* (JD) samples compared with other edible lily samples. The relative contents of *L. regale* (MJ) samples were also listed. Data are presented as the mean ± standard error (SE, $n = 3$). Different lowercase letters indicate statistically significant differences ($P < 0.05$). LY: *L. brownii* var. *viridulum*, SD: *L. pumilum*, LZ: *L. davidii* var. *willmottiae*. F: flavonoids, P: phenolic acids, S: steroid saponins, C: coumarins, O: other compounds. nd: not detected.

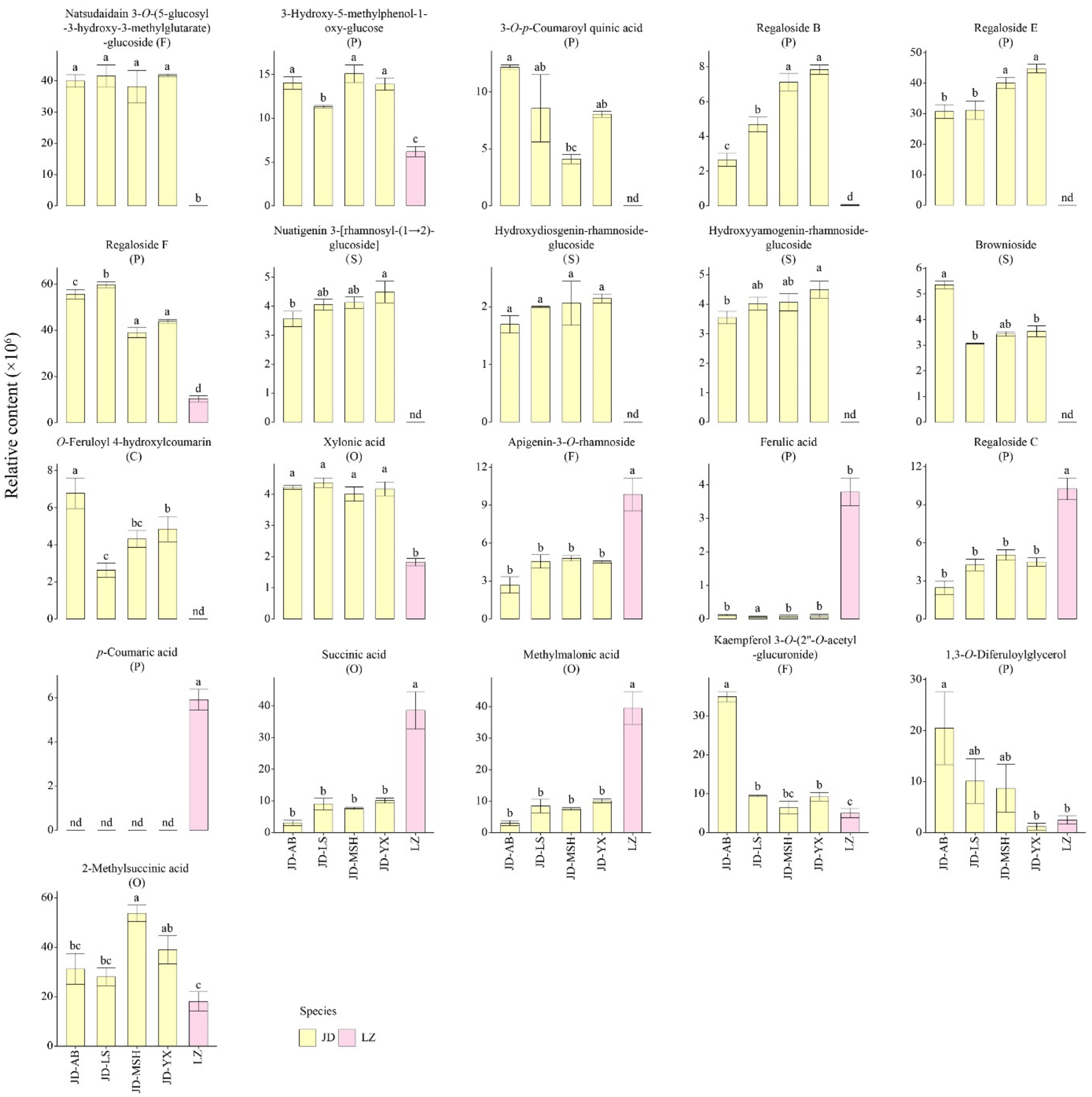

Figure S6. The relative contents of differential metabolites in *Lilium lancifolium* (*Juan Dan*, JD) and *L. davidii* var. *willmottiae* (*Lanzhou Baihe*, LZ) samples. Data are presented as the mean ± standard error (SE, $n = 3$). Different lowercase letters indicate statistically significant differences ($P < 0.05$). F: flavonoids, P: phenolic acids, S: steroid saponins, C: coumarins, O: other compounds. nd: not detected.

Table S1. A list of upregulated metabolites identified between the purple and white lily bulbs.

No.	Compound	Formula	Ionization model	Precursor ions (Q1) (Da)	Molecular Weight (Da)	Class
1	Cyanidin 3-rutinoside	$C_{27}H_{31}ClO_{15}$	$[M-Cl]^+$	595.00	630.11	Flavonoids
2	Hesperetin 5-*O*-glucoside	$C_{22}H_{24}O_{11}$	$[M-H]^-$	463.13	464.11	Flavonoids
3	Apigenin-3-*O*-rhamnoside	$C_{22}H_{24}O_{8}$	$[M-H]^-$	415.10	416.13	Flavonoids
4	Isochrysoeriol C-hexosyl-*O*-hexoside	$C_{28}H_{32}O_{16}$	$[M+H]^+$	625.18	624.17	Flavonoids
5	Isohyperoside	$C_{21}H_{20}O_{12}$	$[M+H]^+$	465.10	464.10	Flavonoids
6	Hyperoside	$C_{21}H_{20}O_{12}$	$[M-H]^-$	463.10	464.08	Flavonoids
7	Spiraeoside	$C_{21}H_{20}O_{12}$	$[M-H]^-$	463.00	464.08	Flavonoids
8	Isoquercetrin	$C_{21}H_{20}O_{12}$	$[M+H]^+$	465.10	464.08	Flavonoids
9	6-Hydroxykaempferol-7-*O*-glucoside	$C_{21}H_{20}O_{12}$	$[M+H]^+$	465.10	464.08	Flavonoids
10	Isorhamnetin-3-*O*-glucoside	$C_{22}H_{22}O_{12}$	$[M+H]^+$	479.11	478.10	Flavonoids
11	Quercetin glu-rha	$C_{27}H_{30}O_{16}$	$[M+H]^+$	611.16	610.15	Flavonoids
12	Rutin	$C_{27}H_{30}O_{16}$	$[M-H]^-$	609.10	610.13	Flavonoids
13	Bioquercetin	$C_{27}H_{30}O_{16}$	$[M-H]^-$	609.10	610.13	Flavonoids
14	Quercetin glu-glu	$C_{27}H_{30}O_{17}$	$[M+H]^+$	627.16	626.15	Flavonoids
15	6-Hydroxykaempferol-3,6-*O*-diglucoside	$C_{27}H_{30}O_{17}$	$[M+H]^+$	627.20	626.12	Flavonoids
16	Methylquercetin glu-rha	$C_{28}H_{32}O_{16}$	$[M+H]^+$	625.18	624.17	Flavonoids
17	Isorhamnetin 3-*O*-neohesperidoside	$C_{28}H_{32}O_{16}$	$[M+H]^+$	625.17	624.14	Flavonoids
18	Quercetin-*O*-feruloyl-pentoside	$C_{30}H_{26}O_{14}$	$[M+H]^+$	611.16	610.15	Flavonoids
19	1-*O*-*p*-Coumaroylglycerol	$C_{12}H_{14}O_{5}$	$[M+H]^+$	239.08	238.08	Phenolic acids
20	1-*O*-Caffeoyl-glucopyranose	$C_{15}H_{18}O_{9}$	$[M-H]^-$	341.08	342.08	Phenolic acids
21	Sinapic acid-hexoside	$C_{17}H_{22}O_{10}$	$[M-H]^-$	385.11	386.12	Phenolic acids
22	Regaloside A	$C_{18}H_{24}O_{10}$	$[M+H]^+$	401.14	400.12	Phenolic acids
23	Regaloside B	$C_{20}H_{26}O_{11}$	$[M+H]^+$	443.15	442.13	Phenolic acids
24	Regaloside C	$C_{18}H_{24}O_{11}$	$[M-H]^-$	415.17	416.13	Phenolic acids
25	26-*O*-glu-3,26-dihydroxy-5-cholesten-16,22-dioxo-3-*O*-rha(1→2)-glucoside	$C_{45}H_{72}O_{18}$	$[M+H]^+$	901.47	900.47	Steroids
26	*O*-Feruloyl 4-hydroxylcoumarin	$C_{19}H_{14}O_{6}$	$[M+H]^+$	339.10	338.07	Coumarins
27	Acanthoside B	$C_{28}H_{36}O_{13}$	$[M-H]^-$	579.21	580.19	Lignans
28	N-Hexosyl-*p*-coumaroyl putrescine	$C_{19}H_{28}N_{2}O_{7}$	$[M+H]^+$	397.10	396.17	Others
29	3-Methyl-2-oxobutanoic acid	$C_{5}H_{8}O_{3}$	$[M-H]^-$	115.00	116.04	Others
30	2-Methylsuccinic acid	$C_{5}H_{8}O_{4}$	$[M-H]^-$	131.04	132.04	Others
